# In the Era of Systematic Reviews, Does the Size of an Individual Trial Still Matter?

**DOI:** 10.1371/journal.pmed.0050004

**Published:** 2008-01-03

**Authors:** Gordon H Guyatt, Edward J Mills, Diana Elbourne

## Abstract

Background to the debate: Systematic reviews that combine high-quality evidence from several trials are now widely considered to be at the top of the hierarchy of clinical evidence. Given the primacy of systematic reviews—and the fact that individual clinical trials rarely provide definitive answers to a clinical research question—some commentators question whether the sample size calculation for an individual trial still matters. Others point out that small trials can still be potentially misleading.

## Gordon Guyatt and Edward Mills' Viewpoint: It Is a Delusion to Think That Sample Size Calculations of Individual Trials Matter

Funding agencies, ethics review boards, journals, and investigators are often preoccupied with power calculations and sample sizes required in clinical trials. We argue that the current practice of sample size justification for randomized clinical trials (RCTs) represents a willing self-deception. Recognizing and adjusting to current realities of RCT conduct may be necessary.

In the high-income nations where most RCTs are organized and funded, chronic diseases are responsible for most morbidity and mortality. In these conditions, multiple pathogenic and behavioral mechanisms determine outcome. Thus, we can anticipate only small to moderate treatment effects from therapies that address only one or two mechanisms. Furthermore, events often occur over a prolonged period of time.

Investigators organizing clinical trials therefore face daunting obstacles. Providing definitive answers in the face of low event rates and small-to-moderate treatment effects necessitates sample sizes in the thousands or tens of thousands. Organizing trials that will enroll such samples involves enormous challenges, as does monitoring the quality of enrollment and data collection once the trials begin. Funding for such mega-trials is very limited, and is often restricted to industry sources.

Even very large trials often produce results that are far from definitive. For instance, the CAPRIE trial (Clopidogrel versus Aspirin in Patients at Risk of Ischemic Events) addressed the relative merits of these two drugs in over 19,000 patients with atherosclerotic vascular disease. The confidence interval around the statistically significant reduction in vascular events with clopidogrel included a lower boundary (0.3% relative risk reduction) that would preclude administration of this expensive drug [1]. Furthermore, the results of even very large trials may prove discrepant with one another [2].

Thus, it is seldom that single trials, even very large ones, provide definitive answers. The scientific community has appropriately accepted that only systematic reviews and meta-analyses combining high-quality evidence from many RCTs will yield robust answers. Individual trials are best viewed as providing important information that contributes to the larger body of evidence.

The clinical trial community responds to these problems with a variety of understandable, pragmatic strategies that nevertheless involve a degree of denial. Investigators typically decide how many patients they can feasibly enroll and then find ways of making assumptions that will justify embarking on a trial with a feasible sample size. These assumptions typically involve choosing a level of delta (the threshold effect below which they are ready to accept a false negative result) that exceeds the minimum effect many patients would consider important.

Other popular strategies are even more problematic. Investigators choose composite endpoints that include a wide range of components that would be important to patients, creating a high risk of misleading interpretation [3]. Investigators focus on outcomes that are more frequent, but less important: for instance, in patients with diabetes, crossing a threshold of serum glucose, or earlier need for a second medication, rather than complications of illness such as major vascular events, neuropathy, or visual impairment.

Perhaps an even more damaging consequence of the unrealistic insistence that individual trials be powered to produce definitive results is that RCTs that would contribute to the body of knowledge are never undertaken. It is unclear how many potential trialists abandon conduct of a trial when they confront its sample size implications, or when they face demands from funding agencies and review committees regarding the sample size they must generate. Our experience in the world of clinical investigation suggests, however, that a large number of potential trials get abandoned. The result is that questions that could ultimately be resolved by a systematic review and meta-analysis remain unanswered, or inadequately answered [4].

How can we resolve this dilemma? Peer-reviewed granting agencies should cease to ask the question “Is this trial powered to definitively answer its primary question?” Rather, they should ask a series of more appropriate alternative questions. First, how important is the issue the investigators propose to address? Are other groups throughout the world investigating the same, or similar questions? How much trial funding is the agency willing to provide? Within the limits of what the agency is willing to fund, have the investigators gone to appropriate lengths to include collaboration that will maximize the number of patients they will be able to enroll?

One could question whether it is ethical to enroll patients in a trial that makes no pretense of definitively answering a question. Indeed, some have asserted that underpowered trials are unethical [5]. But is it not ethical to contribute to a body of knowledge that will ultimately lead to a definitive answer? Is it not unethical to tell patients that a trial will be definitive when that is very unlikely? Is it not more ethical to provide patients with a realistic notion of the contribution of the trial in which they may participate: that it will be one of a number of such studies that will ultimately resolve the issue?

What will result from the conceptual shift we propose? First, agencies and investigators will undertake more RCTs, and evidence for important questions will accumulate more quickly. Second, investigators will be less tempted to stretch their resources and capacities for quality control, and validity of RCTs will improve. Third, when we abandon the current delusion that sample size matters, our minds will open to new strategies for efficiently obtaining crucial evidence for important health issues.

## Diana Elbourne's Viewpoint: Trials That Are Too Small Are Potentially Misleading

When I first started working with clinicians on randomised trials a quarter of a century ago, the most important point I felt I needed to stress was the centrality of randomisation (and allocation concealment) for reducing the very real risks of selection bias at trial entry. Only once that idea was firmly embedded would I consider the many other aspects of trials to which I, as a methodologist, felt I might have something to contribute—such as post-randomisation selection biases, assessment biases, and, of course, sample size. While I would still consider randomisation and allocation concealment the most important issue, sample size doesn't come far behind.

As a statistician, I am often asked, “What size should a trial be? Is 100 patients going to be enough? Or 10? Or 1,000?” Of course, I have to say that there is no absolute number. The considerations may be different if the researcher is thinking about costs, ethics, or about statistical power. Here I will concentrate mainly on the statistical issues.

There may be only one chance (the so-called “window of opportunity”) to conduct a trial, so it needs to be large enough to be able to address the question that is being asked. If one postulates a size of effect that is both likely to be able to influence clinical practice and is feasible, then one wants to have a sample size that is large (powerful) enough to be able to detect that effect with reasonable confidence, allowing for acceptable errors. The corresponding power calculations are simple but the assumptions on which they are based may be problematic. The acceptable errors are traditionally set at 5% (or 1%) for a type 1 error, and 20% (or 10%) for a type 2 error. There is nothing magic about these levels other than that they are generally seen as acceptable risks to run.

Much more problematic are decisions about the effect size. There is always a trade-off between a very large effect that would almost certainly change clinical practice and a more realistic estimate based on emerging empirical evidence (preferably from a systematic review or a pilot study) about the likelihood of a particular effect size. Similarly, effect sizes (at least for categorical variables) take into account the incidence of the primary outcome in the control group. It is a common experience in trials to find that an outcome is less common than estimated, with potential implications for the statistical power.

Power calculations are clearly an inexact science. However, the fact that a process is difficult does not mean we should abandon it—if the notion of an appropriate sample size is useful. The main reason I would still wish trialists and funders to take notice of statistical power is that trials that are too small are potentially misleading. On the one hand they may miss realistic, moderate treatment effects that would be clinically important [6], and a potentially useful intervention may therefore be dismissed. This is particularly worrying if it means that no further trials are carried out because the treatment doesn't look as if it's going to be clinically or commercially valuable. On the other hand, if an underpowered trial does find a statistically significant effect, this is most likely to be a chance finding that over-estimates the size of effect [7]. This occurrence may also stifle further trials as many will feel that it is unethical to randomise if a treatment looks effective (at least in terms of statistical significance).

No trial *should* stand alone. Before a new trial is considered, the existing evidence from systematic reviews (with statistical meta-analysis if appropriate) should be used to inform a decision on whether the new trial is necessary. If it is, then the existing evidence should also be used to help shape the design of the trial, including the sample size. Similarly, once an individual trial is completed, the results should be used to update the systematic review to inform practice and further research.

But systematic reviews cannot take the place of trials. Systematic reviews must be populated by the trials. If, however, the results of a small trial stifle further trials being carried out, then the main strength of a meta-analysis (which is to increase the precision of the estimate of effect) will not be realised. And this is even without considering the issue of publication bias from small trials [8].

Small trials are often (but not invariably) conducted in a single centre. This limits the generalisability of the results to different populations. Again, this is not problematic if there is a systematic review populated by several trials with a range of populations from different settings. But what if a small trial stifles these various trials being conducted?

Finally, I would like to return to the ethics of conducting trials. We are asking prospective trial participants to volunteer to help in an important collaborative venture. We should not be asking them to volunteer if the trial is not likely to provide an answer to the question or may even be misleading [9]. Nevertheless, we would be doing even worse to ask them to take part in a large study aiming to assess effectiveness that was not securely randomised. Size may be important, but bias is worse than imprecision.

## Gordon Guyatt and Edward Mills' Response

Professor Elbourne makes excellent points. Minimizing bias in clinical trials should be paramount. Systematic reviews should inform clinical trials. Investigators should conduct the largest multicenter trials that feasibility permits.

Professor Elbourne is concerned that small trials may mislead. She is correct, particularly if the trial results are substantially biased upward because of poor design or because they stop early for apparent benefit [10]. The latter is a particular problem, because such trials tend to receive undue attention [11].

The problem of misleading small trials is, however, correctable. It is possible to educate the clinical community. Trial design is far better than it was a decade ago. Those designing trials are awaking to the dangers of stopping early for apparent benefit, and clinicians are realizing that systematic reviews of all the high-quality evidence available must guide decision making. Systematic reviews go a long way to ameliorating the misleading inferences that Professor Elbourne fears.

Professor Elbourne is concerned that small trials will stifle investigation. It is an empirical question whether her concerns or ours are more important in discouraging clinical trial conduct. We have experienced few instances when small trial results seriously discouraged investigation, and the strategies we have suggested can deal with those situations. We have, however, experienced dozens of instances in which investigators are caught between the Scylla of power calculations that demand extravagant sample sizes, and the Charybdis of grant committees unwilling to commit the resources required to achieve these sample sizes.

Demanding adequate power to allow a single trial to definitively settle an issue is naïve. Furthermore, stifling of clinical trials by a paradigm that accepts this naïve approach is a present, immediate, and dire threat to accumulating adequate clinical trial evidence to settle important questions. Addressing this problem necessitates the change in the principles underlying funding of clinical trials that we suggest.

## Diana Elbourne's Response

Readers who are looking for a clash between two diametrically opposed views may be disappointed in this exchange—we agree on so much.

We agree on the centrality of minimising bias, and of systematic reviews. We are also optimists (both by nature and with some supportive evidence) and think that there have been improvements in the design, conduct, and reporting of trials, as well as in some aspects of the climate in which they operate—notably the greater acceptance, volume, and quality of systematic reviews, and also the increase in trial registration.

We agree that there is a concern that small trials may mislead and may stifle investigation. The extent of the effect is surely a topic for empirical research when sufficient time in the new trials climate has elapsed.

And we agree investigators should conduct the largest multi-centre trials that feasibility permits. Perhaps, therefore, our main differences are about the feasibility of getting those large trials funded. A recent qualitative study concluded that:

“[A] range of skills and a degree of agility are required for negotiations with potential funders and collaborators…It may also be desirable for [public sector] funding bodies to demonstrate reciprocal skills and flexibility in assessing the financial plans of research applicants.” [12]

As it may be more cost-effective to reap the benefits of scale by conducting a small number of large trials rather than a large number of smaller trials, this agility may include such strategies as providing relatively more funding for pilot and feasibility phases to demonstrate that particular suitably large trials can be conducted, with safeguards for the funding bodies to be able to withdraw support for those trials which are not succeeding.

As an optimist, I believe it is possible to educate not only the clinical community but also the funding bodies so that, as far as possible, researchers, clinicians, and funders can work together with other stakeholders (especially patients) to ensure that suitable trials get funded and produce results that are capable of moving forward the care that patients and their families can receive.

## Abbreviations

RCT: randomized clinical trial
